# Addition of Bevacizumab to Chemotherapy in Advanced Non-Small Cell Lung Cancer: A Systematic Review and Meta-Analysis

**DOI:** 10.1371/journal.pone.0022681

**Published:** 2011-08-02

**Authors:** André Bacellar Costa Lima, Ligia T. Macedo, André Deeke Sasse

**Affiliations:** 1 Departamento de Clínica Médica, Faculdade de Ciências Médicas, Universidade Estadual de Campinas (UNICAMP), Campinas, São Paulo, Brasil; 2 Centro de Evidências em Oncologia (CEVON), Campinas, São Paulo, Brasil; Penn State Hershey Cancer Institute, United States of America

## Abstract

**Introduction:**

Recently, studies have demonstrated that the addition of bevacizumab to chemotherapy could be associated with better outcomes in patients with advanced non-small cell lung cancer (NSCLC). However, the benefit seems to be dependent on the drugs used in the chemotherapy regimens. This systematic review evaluated the strength of data on efficacy of the addition of bevacizumab to chemotherapy in advanced NSCLC.

**Methods:**

PubMed, EMBASE, and Cochrane databases were searched. Eligible studies were randomized clinical trials (RCTs) that evaluated chemotherapy with or without bevacizumab in patients with advanced NSCLC. The outcomes included overall survival (OS), progression-free survival (PFS), response rate (RR), toxicities and treatment related mortality. Hazard ratios (HR) and odds ratios (OR) were used for the meta-analysis and were expressed with 95% confidence intervals (CI).

**Results:**

We included results reported from five RCTs, with a total of 2,252 patients included in the primary analysis, all of them using platinum-based chemotherapy regimens. Compared to chemotherapy alone, the addition of bevacizumab to chemotherapy resulted in a significant longer OS (HR 0.89; 95% CI 0.79 to 0.99; p = 0.04), longer PFS (HR 0.73; 95% CI 0.66 to 0.82; p<0.00001) and higher response rates (OR 2.34; 95% CI 1.89 to 2.89; p<0.00001). We found no heterogeneity between trials, in all comparisons. There was a slight increase in toxicities in bevacizumab group, as well as an increased rate of treatment-related mortality.

**Conclusions:**

The addition of bevacizumab to chemotherapy in patients with advanced NSCLC prolongs OS, PFS and RR. Considering the toxicities added, and the small absolute benefits found, bevacizumab plus platinum-based chemotherapy can be considered an option in selected patients with advanced NSCLC. However, risks and benefits should be discussed with patients before decision making.

## Introduction

Lung cancer affects approximately 200,000 patients in the United States and is the leading cause of cancer-related deaths in both men and women [1]. More than 1.3 million lung cancer patients die annually worldwide. More than 80% of these patients have non–small cell lung cancer (NSCLC) [2], and at least 51% lung cancer patients are diagnosed with metastatic disease. Palliative chemotherapy increases overall survival and quality of life when compared to supportive care as stated in a meta-analysis [3], and these patients have an average survival of 8 to 10 months when treated with platinum-based regimens [4]. Currently, there is no universally accepted standard regimen for first-line treatment of advanced NSCLC, as platinum-based chemotherapy has reached a plateau on survival benefit that is no longer than 10 months, on average.

Agents that target specific pathways in the development or progression of NSCLC have shown useful clinical activity. Vascular endothelial growth factor (VEGF) is a potent endothelial-specific angiogenic factor that is expressed in a wide array of tumors. In NSCLC, high levels of VEGF expression are associated with a poor prognosis [5], suggesting that treatment targeted toward this pathway might be significant therapeutically. Bevacizumab is a monoclonal antibody with a high affinity for VEGF, and thereby prevents its interaction with the VEGF receptor [6].

A randomized phase II trial found that the addition of bevacizumab to carboplatin-paclitaxel improved response rate (RR) (31.5% *vs* 18.8%) and time to progression (7.4 months *vs* 4.2 months) when compared to chemotherapy alone, in patients with advanced NSCLC [7]. There was also a nonsignificant improvement in overall survival (OS). In this trial, patients whose tumors had squamous cell histology were found to be at greater risk for developing hemoptysis. Because of that, in the subsequent trials only patients with predominantly non-squamous NSCLC were studied.

In October 2006, the U.S. Food and Drug Administration granted approval for bevacizumab for use in advanced NSCLC [8], based on data from a phase III trial (E4599) conducted by the Eastern Cooperative Oncology Group (ECOG) [9], [10], which excluded squamous cell histology. This trial compared carboplatin-paclitaxel with and without bevacizumab in 878 patients, and the results indicated a significant improvement in RR (35% *vs* 15%), progression-free survival (PFS) (6.2 *vs* 4.5 months) and OS (12.3 *vs* 10.3 months) related to bevacizumab.

Since there is no standard dose or schedule for bevacizumab in the treatment of lung cancer, a second randomized phase III trial (AVAiL) [11], [12] compared cisplatin-gemcitabine with or without bevacizumab, 7.5 mg/kg or 15 mg/kg, in 1,043 patients with advanced NSCLC. There was a smaller, but still significant improvement in PFS (6.7 *vs* 6.5 *vs* 6.1 months, respectively) and RR (37.8% *vs* 34.6% *vs* 21.6%, respectively) at both doses of bevacizumab, but without a difference in OS (13.6 *vs* 13.4 *vs* 13.1 months, respectively), suggesting that the benefit of bevacizumab could be dependent on the chemotherapy regimen used. It was stated that these differences could be only due to AVAiL being underpowered to detect OS benefits. Indeed, after initiated, the primary endpoint of AVAiL was amended from OS to PFS, following presentation of E4599 results.

As the results of clinical trials were not completely consistent, and none of them was large enough to accurately interpret the efficacy and safety of bevacizumab in combination with chemotherapy, the aim of this meta-analysis was to evaluate and to quantify the effectiveness and safety of bevacizumab in patients with advanced NSCLC.

## Methods

This systematic review was originally completed in the context of an evidence-based training, developed by the Centre for Evidences in Oncology (CEVON) workgroup, in the State University of Campinas (UNICAMP), Brazil. All the evidence was selected and reviewed by two members of CEVON and discussed with the group and the coordinator (ADS). All work produced by CEVON is editorially independent and does not have any funding source.

### Search strategy

A wide search of the main computerized databases of interest was conducted, including PubMed/MEDLINE, EMBASE, LILACS, ClinicalTrials.gov and CENTRAL. The ASCO, ESMO and IASLC Meeting websites were also scrutinized. We used a sensitive search strategy with words related to lung, cancer, chemotherapy, bevacizumab, and randomized trials in all fields. For PubMed/MEDLINE we used the following search terms: (“lung neoplasms”[MeSH Terms] OR (“lung”[All Fields] AND “neoplasms”[All Fields]) OR “lung neoplasms”[All Fields]) AND (“drug therapy”[Subheading] OR (“drug”[All Fields] AND “therapy”[All Fields]) OR “drug therapy”[All Fields] OR “drug therapy”[MeSH Terms]) AND (“bevacizumab”[All Fields]) AND (random*[All Fields]).

All references of relevant articles were scanned and all additional studies of potential interest were retrieved for further analysis. Two reviewers analyzed the list of references and independently selected the studies. The search included literature published or presented up to December 2010.

### Selection criteria

We sought to identify all published randomized controlled clinical trials with a parallel design comparing chemotherapy with or without bevacizumab in patients with advanced NSCLC. To minimize possible bias due to interaction of biologic agents, we excluded trials or arms containing agents targeted against the epidermal growth factor receptor (EGFR).

### Data extraction

The name of the first author and the year of publication of the article were used for identification purposes. Two reviewers independently extracted the data from all included studies. A third reviewer was consulted to resolve disagreements. The outcomes analyzed were OS, PFS, RR, incidence of Common Toxicity Criteria (CTC) scale grade 3/4 toxicities and treatment related mortality.

The hazard ratios (HRs) of time-to-event data (OS and PFS) were directly extracted from the original studies or were estimated indirectly using either the reported number of events and the corresponding p-value for the log-rank statistics, or by reading off survival curves as suggested by Parmar and colleagues [13]. The calculations were carried out using a spreadsheet provided by Tierney and colleagues [14]. For this, the original survival curves from electronic publication were enlarged, and data extraction was based on reading off electronic coordinates for each point of interest in order to decrease reading errors. The number of events and number at risk were abstracted for each dichotomous data comparison evaluated.

### Statistical analysis and synthesis

Details regarding the main methodological dimensions empirically linked to bias as described by Deeks and colleagues [15] were extracted, and the methodological quality of each selected trial were assessed by two reviewers (ABL and LTM). These data were combined in a pre-specified subgroup, and sensitivity analyses were performed to test the stability of our conclusions.

All meta-analyses were performed using Review Manager 5 (RevMan 5; The Nordic Cochrane Centre, The Cochrane Collaboration, Copenhagen, Denmark) with a random effects model. Time-to-event outcomes were compared using an HR. Dichotomous data were compared using an odds ratio (OR). Respective 95% confidence intervals (CI) were calculated for each estimate and presented in forest plots. The pooled HR or OR, symbolized by a solid diamond at the bottom of the forest plot (the width of which represents the 95% CI) is the best estimate of the true (pooled) outcome. The effect of the treatment for each single study was expressed as a ratio of the bevacizumab chemotherapy arm over the chemotherapy alone arm.

Statistical heterogeneity in the results of the trials was assessed by the chi-square test [16], and was expressed by the I*_2_* index, as described by Higgins and colleagues [17]. When considerable heterogeneity was detected (I*_2_*>35%), a possible explanation for it was pursued. When a reasonable cause was found, a separate analysis was performed. Publication bias was evaluated with the Egger's test [15].

## Results

**Figure 1:**
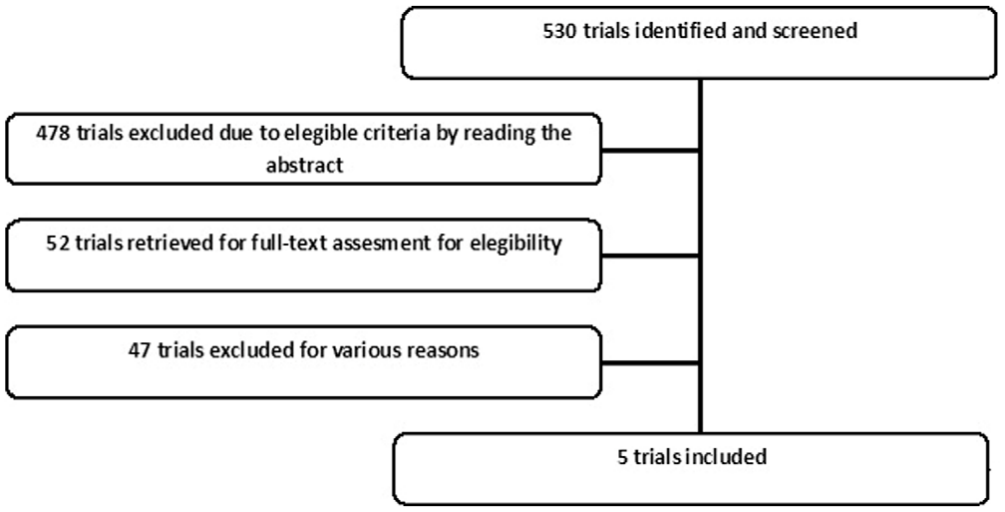
Flow of identification and inclusion of trials.

Our systematic search screened 530 trials, and found six publications related to five randomized clinical trials (2,252 patients) that compared chemotherapy with or without bevacizumab [7], [9], [11], [18]–[20]. Only the most updated data were included in the analysis. Other potential eligible studies were single-armed or involved EGFR inhibitors and were therefore excluded. A diagram represents the flow of identification and inclusion of trials (Figure 1), as recommended by the PRISMA statement [21].

Three phase II and two phase III trials were included in the analysis. All but Herbst et al [19] evaluated first-line palliative therapy. Herbst et al included patients who had progressed during or after one platinum-based regimen. Details about methodology potentially linked to bias are described in Table 1. Crossover was not permitted in the two major phase III trials. Johnson et al's phase II trial [7] included patients with squamous cell histology. As a result of the prohibitive bleeding toxicity in this group, squamous cell histology became an exclusion criterion in the other trials of this meta-analysis.

**Table 1:** Methodological details potentially related to bias.

Authors	Year	Phase	Blindness	Withdrawn description	Alpha error	Beta error	ITT analysis	Multicenter	Sponsor
Johnson et al	2004	II	No	Yes	Yes	Yes	Yes	Yes	Industry
Sandler et al	2006	III	No	Yes	Yes	Yes	No	Yes	Industry
Herbst et al	2007	II	Unclear	Yes	No	No	Yes	Yes	Industry
Reck et al	2009	III	Yes	Yes	Yes	Yes	Yes	Yes	Industry
Nishio et al	2009	II	No	Yes	No	No	No	Yes	No report

ITT: intention-to-treat analysis.

Johnson et al excluded one patient in the experimental group because of the discovery of CNS metastasis. E4599 excluded 28 patients because of eligibility violations. One patient in the experimental group in the Herbst et al trial did not receive bevacizumab, although included in the analysis. All patients in the AVAiL trial were included in the primary analysis despite 57 patients receiving no study therapy as a result of eligibility violations, consent withdrawal, adverse events (AEs) and other reasons. In the present meta-analysis we used the intention-to-treat (ITT) data from the trials, when possible. It was not possible to extract ITT data in E4599 trial, which excluded ineligible patients from primary analysis. ITT data from Nishio et al was not available either, since data was extracted from meeting presentations.

Chemotherapy regimens included paclitaxel plus carboplatin up to six cycles [7], [9], [20], cisplatin plus gemcitabine up to six cycles [11], and single agent docetaxel or pemetrexed until disease progression [19]. Drugs were administered on the first day of each 3-week cycle. The dose of bevacizumab was 7.5 mg/kg [7], [12] or 15 mg/kg [7], [9], [12], [19], [20] on day 1 of each cycle. Particular features of all trials are described in Table 2.

**Table 2:** Description of interventions and patients included.

Author/year	Study/arm	Patients enrolled	Setting	Primary endpoint	ECOG 0, 1(%)	Histology	Maintenance of bevacizumab (maximum cycles)	Crossover permitted
Johnson 2004	TP	32	1^st^ line	PFS	93.7	NSCLC	Yes (18)	Yes
	TP+Bev (7.5)	32			96.8			
	TP+Bev (15)	35			88.5			
Sandler 2006	TP	444	1^st^ line	OS	100	Non-squamous NSCLC	Yes (until disease progression)	No
	TP+Bev (15)	434			100			
Herbst 2007 *	D or P	41	2^nd^ line	PFS	97.6	Non-squamous NSCLC	Yes (until disease progression)	Yes
	D or P+Bev (15)	40			100			
Reck 2009	GP	347	1^st^ line	PFS	100	Non-squamous NSCLC	Yes (until disease progression)	No
	GP+Bev (7.5)	345			100			
	GP+Bev (15)	351			100			
Nishio 2009	TP	59	1^st^ line	PFS	NR	Non-squamous NSCLC	Yes (until disease progression)	NR
	TP+Bev (15)	121						

NR: no report; GP: gemcitabine 1,250 mg/m^2^ plus cisplatin 80 mg/m^2^; TP: paclitaxel 200 mg/m^2^ plus carboplatin AUC 6; D: docetaxel 75 mg/m^2^; P: pemetrexed 500 mg/m^2^; Bev (7.5): bevacizumab 7.5 mg/kg. Bev (15): bevacizumab 15 mg/kg.

*Included patients that had progressed after one platinum-based regimen.

### Overall Survival

The impact of bevacizumab treatment on OS was extracted directly or estimated indirectly from published data of four trials included in this review (2,072 patients). Nishio et al's trial had immature data, due to short duration of follow-up, and did not presented OS results.

**Figure 2:**
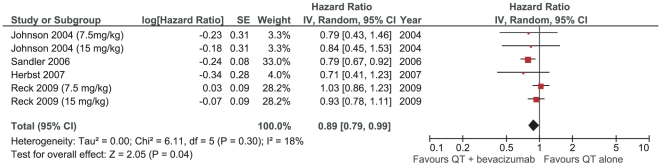
Meta-analysis of overall survival.

In the meta-analysis, the HR for OS favored bevacizumab combination chemotherapy [HR 0.89 (0.79–0.99), p = 0.04], without significant heterogeneity between studies (I^2^ = 18%; p = 0.30) (Figure 2). This result indicates that there is a slight but significant reduction in mortality (11%) with the addition of bevacizumabe to chemotherapy.

Evaluating only trials studying first-line therapy, the meta-analysis showed similar results, however without statistical significance [HR 0.90 (0.79–1.01), p = 0.08]. We also calculated the absolute prolongation of survival for patients who were treated with first-line chemotherapy plus bevacizumab compared with patients with a predicted survival on platinum-based doublets of 8 months [4]. The absolute benefit of association of bevacizumab in median survival in first line therapy was estimated in 26 days (0.88 months).

The sensitivity analyses performed using subgroups linked to methodological aspects confirmed similar results. Funnel plots did not show evidence of significant publication bias risk.

### Progression free survival

**Figure 3:**
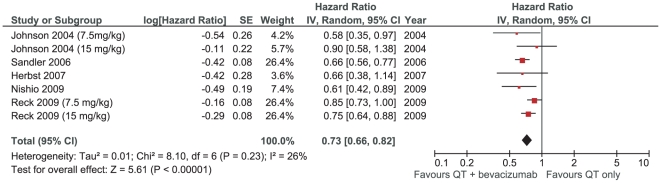
Meta-analysis of progression-free survival.

Progression-free survival was the primary endpoint in four trials. The meta-analysis showed a significant benefit related to bevacizumab [HR 0.73 (0.66–0.82), P<0.00001]. Again, there was no significant heterogeneity between trials (I_2_ = 26%; p = 0.23) (Figure 3). Assuming a median PFS of 4 months for patients in first-line therapy with platinum-based doublets, we estimated an absolute benefit of 1.4 months for association of bevacizumab to chemotherapy.

### Overall response rates

**Figure 4:**
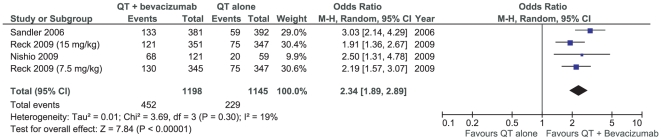
Meta-analysis of overall response rates in first-line therapy (non-squamous NCSLC).

The results concerning the overall response rates (RR) showed high heterogeneity between trials (I_2_ = 53%; p = 0.06). The inclusion of one trial evaluating bevacizumab added to second-line therapy could be the cause of this heterogeneity. It might also be due to the disparate distribution of squamous cell histology patients in the baseline data of the Johnson et al trial and difficult response evaluation due to an early toxicity. In fact, pooling the data only from trials evaluating first-line therapy in non-squamous NSCLC, meta-analysis showed a less heterogeneous result (I_2_ = 19%; p = 0.30), and a significant RR favoring the bevacizumab group [OR 2.34 (1.89–2.89), p<0.00001] (Figure 4).

### Toxicities and safety

**Figure 5:**
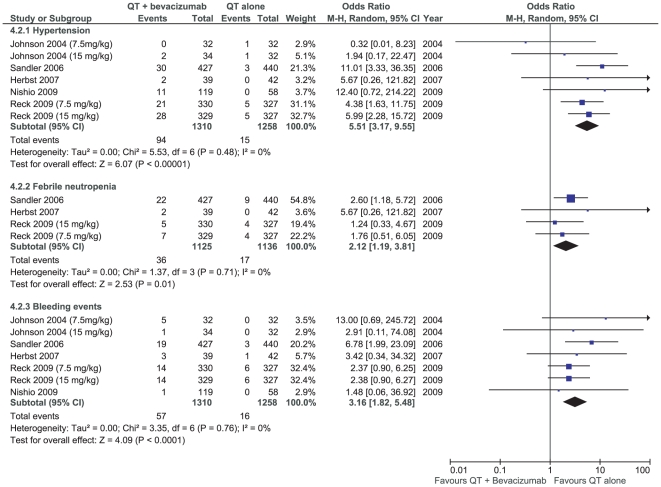
Meta-analysis of grade 3/4 toxicities.

All studies described some sort of toxicity, however, only some of the data were consistently described in the articles. Some of the more clinically relevant grade 3/4 AEs increased by the addition of bevacizumab to chemotherapy were hypertension [OR, 5.51 (3.17–9.55), p<0.00001], bleeding events [OR 3.16 (1.82–5.48), p<0.0001] and febrile neutropenia [OR 2.12 (1.19–3.81), p = 0.01], all presented in Figure 5.

**Figure 6:**
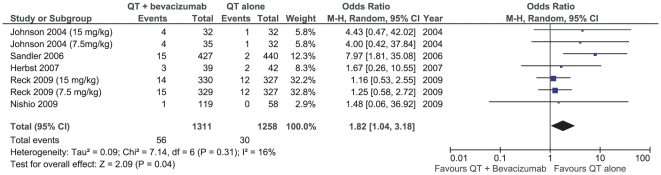
Meta-analysis of deaths related to treatment.

Most important, there was a significant increase in deaths related to treatment associated with the addition of bevacizumab [OR 1.82 (1.04–3.18), p = 0.04] (Figure 6). Most of the deaths in the bevacizumab group were related to bleeding events, neutropenia complications and thromboembolic events. The available data did not provide the opportunity to quantify and to compare each cause of mortality individually.

## Discussion

The main finding of the present meta-analysis is the homogeneous OS improvement provided by the addition of bevacizumab to chemotherapy when compared to chemotherapy alone in the advanced NSCLC setting, with an 11% reduction in risk of death, but with an estimated absolute benefit of less than 1 month in median survival.

The meta-analysis showed also that the addition of bevacizumab to chemotherapy resulted in a significant improvement in both PFS (absolute benefit of 1.4 months in median) and RR (absolute difference of 16%). The benefits were consistent and seemed to be applicable to all patients with non-squamous NSCLC. However, these outcomes can be considered less important to patients, and should be validated with quality of life analysis in order to prove a clear clinical benefit.

Comparing the two phase III trials included in this systematic review, the E4599 trial demonstrated an important OS benefit (HR 0.79, p = 0.003), while AVAiL failed in this goal (HR 0.93, p = 0.420; HR 1.03, p = 0.761; for 7.5 mg/kg and 15 mg/kg groups, respectively). The authors of the AVAiL trial justified the results pointing out the greater use of efficacious second-line therapies, and the slightly more favorable prognostic features in baseline data compared with the E4599 trial. Moreover, the difference in OS between the E4599 and AVAiL original publications was initially explained by preclinical findings showing that paclitaxel induces circulating endothelial progenitor cells (CEPs) whereas gemcitabine does not, and that the addition of an anti-VEGFR2 antibody acts synergistically only in combination with CEP-mobilizing chemotherapeutic agents [22]. However, these findings were generated in preclinical tumor models, and have not been confirmed in clinical trials.

In fact, the absence of heterogeneity between the results in OS meta-analysis suggests that the AVAil trial was not powered to find the small difference obtained in OS. The results suggest that the addition of bevacizumab to cisplatin-based chemotherapy slightly prolongs OS of advanced NSCLC patients, independently of the regimen used.

The present study has the typical limitations of the meta-analytical methodology. Our findings and interpretations were limited by the quality and quantity of data available. An analysis of individual patient data would be more powerful to confirm our findings. Another source of concern is the possible existence of some unpublished studies, which could lead to potential publication bias. However, we found no indication of such bias by using statistical methods designed to detect it.

The toxicities added by bevacizumab, including fatal adverse events are a great concern. A recent systematic review with meta-analysis of 16 trials found that the addition of bevacizumab to chemotherapy, compared with chemotherapy alone, was associated with increased treatment-related mortality, in patients with a variety of advanced solid tumors [23].

The risk of severe toxicity in patients with lung neoplasms may be particularly increased in elderly patients, as stated in an unspecified retrospective analysis of E4599 [24]. Nevertheless, a subanalysis of the safety and efficacy of bevacizumab in 610 elderly (>65 years) patients in SAiL, a large phase IV trial with 2,172 patients, showed no significant difference in AEs and outcomes in this subgroup [25].

One of the most prominent yet reversible AEs related to bevacizumab was hypertension, which was reported to be somewhat manageable [26]. The VEGF antagonism decreases nitric oxide production and leads to constriction of the vasculature and a reduction in sodium ion renal excretion, which ultimately leads to increased blood pressure [27], [28]. Hypertension may also be a consequence of vascular rarefaction, caused by the inhibition of angiogenic growth factors required to construct new capillaries and recruit endothelial progenitor cells [26], [29]. An interesting subset analysis of E4599 suggested that hypertension onset during treatment with bevacizumab may be associated with improved outcomes [30]. This trend was also observed in SAiL phase IV trial [31]. However, predictive biomarkers for response are not yet available for bevacizumab.

In three more recently analyzed studies, patients with squamous cell carcinoma, or a history of therapeutic anticoagulation, hemoptysis, or brain metastases were excluded to minimize the risk of pulmonary or intracerebral hemorrhage, based on results from Johnson et al. Although bleeding events are a concern, severe pulmonary hemorrhage was an uncommon event, as confirmed by the SAiL study [32], [33]. Preliminary data from ARIES (6.1 month median follow-up), a large observational cohort study that comprised 1,031 patients, also suggest a poor correlation between centrally located tumor or presence of any cavitation and higher risk of pulmonary hemorrhage [34]. A recent retrospective exploratory analysis by Besse et al [35] concluded that patients with CNS metastases are at similar risk of developing cerebral hemorrhage, independent of bevacizumab therapy. In fact, ARIES showed that none of the 67 patients with brain metastasis at baseline developed CNS hemorrhage [34]. In the SAiL and ARIES trials, there was no increase in bleeding in patients receiving concurrent bevacizumab and fulldose anticoagulation therapy.

Notably, our study showed a small increase in risk of treatment-related death, in patients receiving the association of bevacizumab to chemotherapy. The difficulty in find a pre-established group of patients at great risk of serious adverse events could be challenging, in clinical practice. Based in recent evidence, all patients treated with bevacizumab should be monitored carefully for bleeding, gastrointestinal tract perforation, and neutropenia.

In conclusion, this meta-analysis demonstrates that bevacizumab combined with standard platinum-based chemotherapy doublets in the first-line setting leads to a small but significantly improved OS, PFS and RR for patients with advanced non-squamous NSCLC. Taking into account the toxicities added and the small increase in risk of treatment-related death, bevacizumab plus platinum-based chemotherapy can be considered an option in selected patients with advanced NSCLC. However, benefits and risks should be discussed with patients before decision making.
